# Comparing commercial pharmacogenetic testing results and recommendations for antidepressants with established CPIC guidelines

**DOI:** 10.3389/fphar.2024.1500235

**Published:** 2024-11-25

**Authors:** Tiffany T. Nguyen, Emili J. W. Leary, Joshua T. Lee, Sanjay K. Shukla, Sara A. Griesbach

**Affiliations:** ^1^ Clinical Pharmacy Services, Marshfield Clinic Health System, Marshfield, WI, United States; ^2^ Center for Precision Medicine Research, Marshfield Clinic Research Institute, Marshfield Clinic Health System, Marshfield, WI, United States

**Keywords:** pharmacogenomics, pharmacogenetic testing results, pharmacogenetic testing company, binning, pharmacogenetics, pharmacogenetic testing, SSRI

## Abstract

**Introduction:**

Increasingly, pharmacogenetic testing helps providers with medication selection based upon patient-specific DNA results. While several government-funded organizations work towards consensus and standardization for testing and interpretation, compliance to these best practices remains inconsistent. Pharmacogenetic testing companies often develop proprietary practices for interpreting and reporting, which can lead to incongruency of reported results among companies and potential discrepancies in interpretation.

**Methods:**

To identify the differences of commercial pharmacogenetic testing vendors’ interpretation of genotype-to-phenotype translations and medication recommendations from the Clinical Pharmacogenetic Implementation Consortium (CPIC) guidelines, a retrospective manual chart review was completed in a large rural healthcare system that utilizes two institution-approved pharmacogenetic vendors. One hundred patients were evaluated: 50 who completed testing through Company A and 50 who completed testing through Company B. Genes of interest for genotype-to-phenotype translation included *CYP2B6*, *CYP2C19*, and *CYP2D6*. Comparison of medication recommendations for drug-gene pairs sertraline (*CYP2B6* and/or *CYP2C19)*, escitalopram (*CYP2C19*), and paroxetine (*CYP2D6*) were compared with recommendations from CPIC, with consideration of the CPIC Serotonin Reuptake Inhibitor Antidepressants (SSRI) guideline 2023 update. This was accomplished via a novel binning process to enable comparison of company-provided binned medication recommendations with CPIC guideline recommendations. Briefly, the binning system included three categorizations based upon the relevant CPIC guideline recommendations–no action needed (green), recommend monitoring (yellow) and therapeutic intervention or alternative recommended (red).

**Results:**

There were 32/250 (12.8%) genotype-to-phenotype translation discrepancies from CPIC guidelines, all from Company A. Of 266 evaluated binned medication recommendations, there were 114 (42.9%) discrepancies between the pharmacogenetic testing companies (Company A: 93 discrepancies, Company B: 21 discrepancies) and CPIC’s guideline based upon comparison with the novel binning system.

**Discussion:**

Significant differences were observed between testing companies’ interpretations and recommendations, which is concerning as these discrepancies could lead to providers making medication decisions that are not supported by CPIC’s clinical practice guidelines. This may result in suboptimal outcomes for patients, leading to patient and provider dissatisfaction and erosion of trust with pharmacogenetic testing. A proposed resolution for the discrepancies in company-to-company interpretation is adherence to the CPIC guidelines and transparency in interpretation practices.

## 1 Introduction

In recent years, providers have increasingly turned to pharmacogenetic testing for assistance with drug optimization, specifically to improve medication use for a given drug-gene pair, which is a known pairing of a gene and drug that may have a potential impact on the pharmacokinetics or pharmacodynamics of that drug. Pharmacogenetic testing is a genetic analysis that identifies changes, or variants, in a given gene; a drug-gene interaction (DGI) occurs when a specific variant in that gene is identified within an individual, thus suggesting a change in the therapeutic effect of the drug due to the variant. As many commercial pharmacogenetic testing companies offer services, evaluating vendors for interpretation practices of drug-gene pairs and drug-gene interaction practices can be challenging. Regulations regarding clinical validity do exist for laboratory developed tests (LDTs)—arguably the most common platform used by pharmacogenetic testing—however, this regulation has not been tightly standardized nor enforced historically (Bousman et al., 2019). The Food and Drug Administration (FDA) is currently revising their regulatory approach of “enforcement discretion” for LDTs, though the proposed changes and implications continue to evolve (Food and Drug Administration FDA, 2024).

Efforts to standardize and guide clinical use of pharmacogenetics have been ongoing by reputable National Institutes of Health (NIH)-supported stakeholders including CPIC, the Pharmacogenomics Knowledgebase (PharmGKB), the Pharmacogene Variation (PharmVar) Consortium, as well as professional organizations like the Association for Molecular Pathology (AMP) and others. The FDA has also engaged in supporting drug-gene pairs with various resources, most notably the Table of Pharmacogenetic Associations. Briefly, CPIC is an international organization that adheres to the Institute of Medicines standards for developing trustworthy clinical practice guidelines with rigorous authorship criteria and management of conflicts of interest (Caudle, 2014). Their goal is to guide healthcare professionals on the evidence-based *use* of pharmacogenetic testing (Relling and Klein, 2011; Gaedigk, 2020). PharmGKB collects, curates, and summarizes drug-gene pairs and genotype-to-phenotype relationships, as well as hosts CPIC and other professional pharmacogenetic guidelines. PharmVar serves as a repository for the definition of consensus variants defining particular alleles for a given pharmacogene, and AMP is a not-for-profit organization whose pharmacogenetic workgroup has developed standards for the minimum sets of variants to query for pharmacogenetic testing panels. Importantly, all of the NIH-funded organizations grade and rate the level of evidence of recommendations according to transparent criteria–CPIC utilizes levels of evidence (high, moderate, weak; Levels of Evidence, 2010) in linking genotype-to-phenotype and provides strength of association recommendations (strong, moderate, optional; Strength of Recommendations, 2011) for therapeutic recommendations. PharmGKB uses an annotation scoring system for both clinical annotations and variant annotations (Level 1A, 1B, 2A, 2B, 3 and 4), and PharmVar utilizes levels of evidence for the allele-haplotype definition reported, however, it should be noted that the “evidence level is not a measure of the quality of the data, but rather reflects the amount and nature of the data a haplotype definition is based upon” (Whirl-Carrillo et al., 2021; Pharmacogene Variation Consortium, 2024). The FDA hosts several resources for drug-gene interactions in the Table of Pharmacogenetic Associations, Table of Pharmacogenomic Biomarkers in Drug Labeling, and, when relevant, in the prescribing information for specific medications. Notably, the source of the information provided by the FDA is not always transparent, reflective of the fact that this information is provided to the FDA by the drug manufacturer which is often not reflected in the primary literature (Pritchard et al., 2022).

All these resources are critically important to standardizing pharmacogenetic testing such that the results may be used to provide evidence-based clinical interventions that are generally free from commercial bias. To this end, the AMP’s Pharmacogenetic Working Group has developed consensus recommendations for alleles that all pharmacogenetic laboratory groups should test: Tier 1 are the fundamental alleles that all platforms should include at minimum, and Tier 2 are recommended supplemental alleles (Pharmacogenomics Knowledgebase, 2024). While AMP’s tiering of recommended alleles do not have associated levels of evidence *per se*, they do offer very clear criteria on what constitutes assignment to Tier 1 or Tier 2, and are generally reliant on the aforementioned NIH-supported organizations (Pratt, 2018). Currently, seven genes have AMP guidelines, though more are anticipated. Around the same time these recommendations were published, several articles identifying variability regarding gene and allele evaluation between companies were also reported (Bousman and Dunlop, 2018; Bousman et al., 2019). This may be attributable to the use of proprietary algorithms and interpretation practices by these testing companies. Of note, currently no enforcing body requires adherence to these standardization practices, thus discrepancies may persist despite these available resources.

While seemingly innocuous, variable interpretation possesses tremendous impact on therapy: medication recommendations may not depend on the patient’s findings but rather the company’s proprietary multigene algorithm. Notably, no proprietary multi-gene algorithm has been validated or endorsed by either the FDA, or by CPIC. This is not the first time this topic has been reported. Discrepancies between testing companies versus CPIC with respect to genotype-to-phenotype translation has been recently documented (Blazy et al., 2022) and previous research has characterized variability in medication recommendations based upon specific genotypes as differing from company to company (Bousman and Dunlop, 2018). Many laboratories use proprietary practices when reporting pharmacogenetic results and making recommendations (Bousman and Dunlop, 2018; Bousman et al., 2019; Luvantseren et al., 2020). As such, the FDA issued warnings to patients and providers (in 2018), and eventually to pharmacogenetic testing companies themselves (in 2019) regarding their medication recommendations based upon genetic findings, citing that the FDA was not aware of credible data supporting those recommendations (Food and Drug Administration FDA, 2018; Food and Drug Administration FDA, 2019). Pharmacogenetic testing companies generally do not publish data on the clinical validity or utility of their algorithms, and when they do, the methodological limitations, failure to find durable clinical outcomes, or lack of robust findings may diminish the strength of their conclusions (Bousman and Dunlop, 2018; Zeier et al., 2018; Greden et al., 2019; Oslin et al., 2022). Pharmacogenetic results interpreted through the lens of proprietary ‘combinatorial’ algorithms may influence providers’ decisions and lead them to diverge from evidence-based guidelines (Vande Voort et al., 2022). A lack of transparency in these algorithms and resulting recommendations has been previously and aptly described as a “Black Box” for pharmacogenetic clinical decision support that poses a potential unrecognized risk to users: are the recommendations clinically valid (Bousman and Eyre, 2020)? While the importance of the genotype-to-phenotype translation and subsequent medication recommendation discrepancies may not be immediately apparent, divergence from reputable, evidence-based recommendations free of conflicts of interest have the potential to negatively impact pharmacogenetic test use: at best, proprietary recommendations may misrepresent drug-gene interactions, eroding clinician’s trust in and/or satisfaction with pharmacogenetics, and at worst they have the potential to cause patient harm via use of lower quality data in making therapeutic recommendations that providers may act upon.

This study aimed to build and expand upon previous reports of medication recommendation discrepancies compared to CPIC guidelines with the use of a novel binning system designed to reflect three levels of potential DGIs for comparison to proprietary algorithm binning.

## 2 Methods

### 2.1 Study design and participants

A retrospective manual chart review was completed for a random sampling of patients who had an order for pharmacogenetic testing placed between 1 July 2018 and 31 December 2023 as part of a pharmacy post-graduate 1-year residency project. The study evaluated eligible patients tested by one of two pharmacogenetic testing vendors approved by the institution: GeneSight™ (Company A) and OneOme™ (Company B), which both companies used deletion/duplication detection and targeted variant analysis as their methodology of analysis. The only inclusion criterion was completion of pharmacogenetic testing (which required test ordering, specimen collection and analysis, and reporting of results) and report availability in the medical record within the specified dates. Patient age was not an exclusion criterion, as the genotype would not vary based upon age, and the medication recommendations could be expected to be translatable to the pediatric population (Bousman et al., 2023). Potential patients meeting the above inclusion criteria were identified and provided by a data analyst and/or pharmacist in an incremental fashion (e.g., blocks of 50 patients to screen) until the sufficient number of patients were identified for each group. To provide an equal and representative sampling, 50 patients per pharmacogenetic testing company were collected: 25 patients tested before the CPIC Serotonin Reuptake Inhibitor Antidepressants (SSRI) 2023 guideline update, and 25 patients tested after. Patient pharmacogenetic results were reviewed by a pharmacist with specific training and experience in pharmacogenetics. CPIC guidelines were used to identify actionable alleles, genotypes and phenotypes for *CYP2B6, CYP2C19*, and *CYP2D6* genotype-to-phenotype translations. Regarding drug-gene interactions, an internal three-tier binning system was developed based upon guidance provided in the CPIC SSRI guideline (Table 1). This was used to compare pharmacogenetic testing companies’ medication recommendations to those of CPIC (Table 2). The protocol was reviewed and deemed exempt as quality improvement by the Marshfield Clinic Institutional Review Board.

**TABLE 1:** Definitions and general recommendations for binning system categories.

	Drug-gene interaction (DGI) level of impact	DGI level of impact definition	General recommendation	Example wording used in CPIC's recommendations
Bin 1 (Green)	No DGI/Unknown DGI/No recommendation	DGI does not significantly affect medication metabolism and/or no indicated increased risk of adverse reaction or loss of efficacy	Continue with FDA recommended dosing	“Initiate therapy with recommended starting dose” “No recommendation due to lack of evidence”
Bin 2 (Yellow)	Moderate DGI	DGI moderately affects medication metabolism and/or indicates a slight increased risk of adverse reaction or loss of function	Initiate at normal starting dose and monitor with or without further dose adjustment	“Initiate therapy with recommended starting dose. Consider a slower titration schedule and lower maintenance dose than normal metabolizers.”
Bin 3 (Red)	Major DGI	DGI significantly affects metabolism and/or indicates an elevated risk of adverse reaction or loss of efficacy	Avoid medication and/or dose adjustment is warranted	“Consider a clinically appropriate antidepressant not predominantly metabolized by CYP2C19. If citalopram or escitalopram are clinically appropriate, consider a lower starting dose, slower titration schedule, and 50% reduction of the standard maintenance dose as compared with normal metabolizers” “Select alternative drug not predominately metabolized by *CYP2D6*.”

**TABLE 2:** Binning system recommendations for the SSRI drug-gene pairs The novel binning system utilizes 3 color-coded bins assigned according to the drug-gene-phenotype pairing recommendations from the CPIC SSRI Guideline with consideration of the guideline update: bin 1 (green; no interaction–no action needed), bin 2 (yellow; moderate interaction–recommend to monitor), and bin 3 (red; major interaction–recommend alternative therapy or dose modification).

	UM	RM	NM	IM	PM
CPIC guidelines before 4/10/2023					
Sertraline and *CYP2C19*	Bin 2	Bin 2	Bin 1	Bin 1	Bin 3
Escitalopram and *CYP2C19*	Bin 3	Bin 3	Bin 1	Bin 1	Bin 3
Paroxetine and *CYP2D6*	Bin 3	NA	Bin 1	Bin 1	Bin 3
CPIC guidelines on or after 4/10/23					
Sertraline and *CYP2B6*	Bin 1	Bin 1	Bin 1	Bin 2	Bin 2
Escitalopram and *CYP2C19*	Bin 3	Bin 2	Bin 1	Bin 2	Bin 3
Paroxetine and *CYP2D6*	Bin 3	NA	Bin 1	Bin 2	Bin 3

Abbreviations: UM, ultrarapid metabolizer; RM, rapid metabolizer; NM, normal metabolizer; IM, intermediate metabolizer; PM, poor metabolizer; NA, not applicable.

### 2.2 Objectives

The objectives of this quality improvement project were to evaluate differences in (1) the pharmacogenetic testing companies’ genotype-to-phenotype translation and (2) behavioral health medication recommendation discrepancies between pharmacogenetic testing companies and CPIC’s guideline.

### 2.3 Novel binning process

Pharmacogenetic testing companies may have their own criteria for categorizing the level of impact of the DGI within patient reports. These are often binned into three categories, color-coded using the traffic light convention: green, for no significant interaction; yellow, for moderate interaction; and red, for major interaction. Our institution created a novel binning system to enable comparison of the pharmacogenetic testing company’s binned medication recommendations to medication recommendations from the CPIC guidelines. This novel binning system also followed the traffic light convention and, aligning with CPIC’s guidelines, sorted each recommendation based on the level of impact of the DGI, allowing for easy comparison to testing companies (Tables 1, 2). Briefly, Bin 1 (green) included CPIC recommendations that did not deviate from standard dosing. Bin 2 (yellow) included recommendations in which standard dosing still applied as the starting dose, however additional monitoring or dose adjustment could be warranted if side effects or efficacy concerns arise. Bin 3 (red) included recommendations which either advise to avoid the drug entirely or recommend a dose adjustment at initiation with additional monitoring.

As behavioral health medications were reported by both pharmacogenetic testing companies and are included in CPIC guidelines, escitalopram, paroxetine and sertraline were selected as the basis of medication comparison. Bins were determined for drug-gene pairs: sertraline and *CYP2C19* (prior to guideline update) or *CYP2B6* (after the guideline update), escitalopram (*CYP2C19*), and paroxetine (*CYP2D6*) based upon CPIC’s SSRI Antidepressants guidelines (Bousman et al., 2023; Hicks et al., 2015) relevant at the time of the pharmacogenetic report date. Of note, sertraline’s recommendations post-guideline update depended upon both *CYP2B6* and *CYP2C19*. For post-guideline update patients, *CYP2C19* had to be wildtype (e.g., normal metabolizer) for comparison as to not confound the *CYP2B6*-related recommendation for sertraline. Patient results were reviewed for commercial laboratory-determined bin recommendations for the three drug-gene pairs (e.g., sertraline/*CYP2B6* or *CYP2C19*, escitalopram/*CYP2C19*, and paroxetine/*CYP2D6*), which were then compared to the novel binning system for each drug-gene pair for discrepancies.

### 2.4 Timeline of CPIC’s Serotonin Reuptake Inhibitor Antidepressants guideline

**FIGURE 1:**
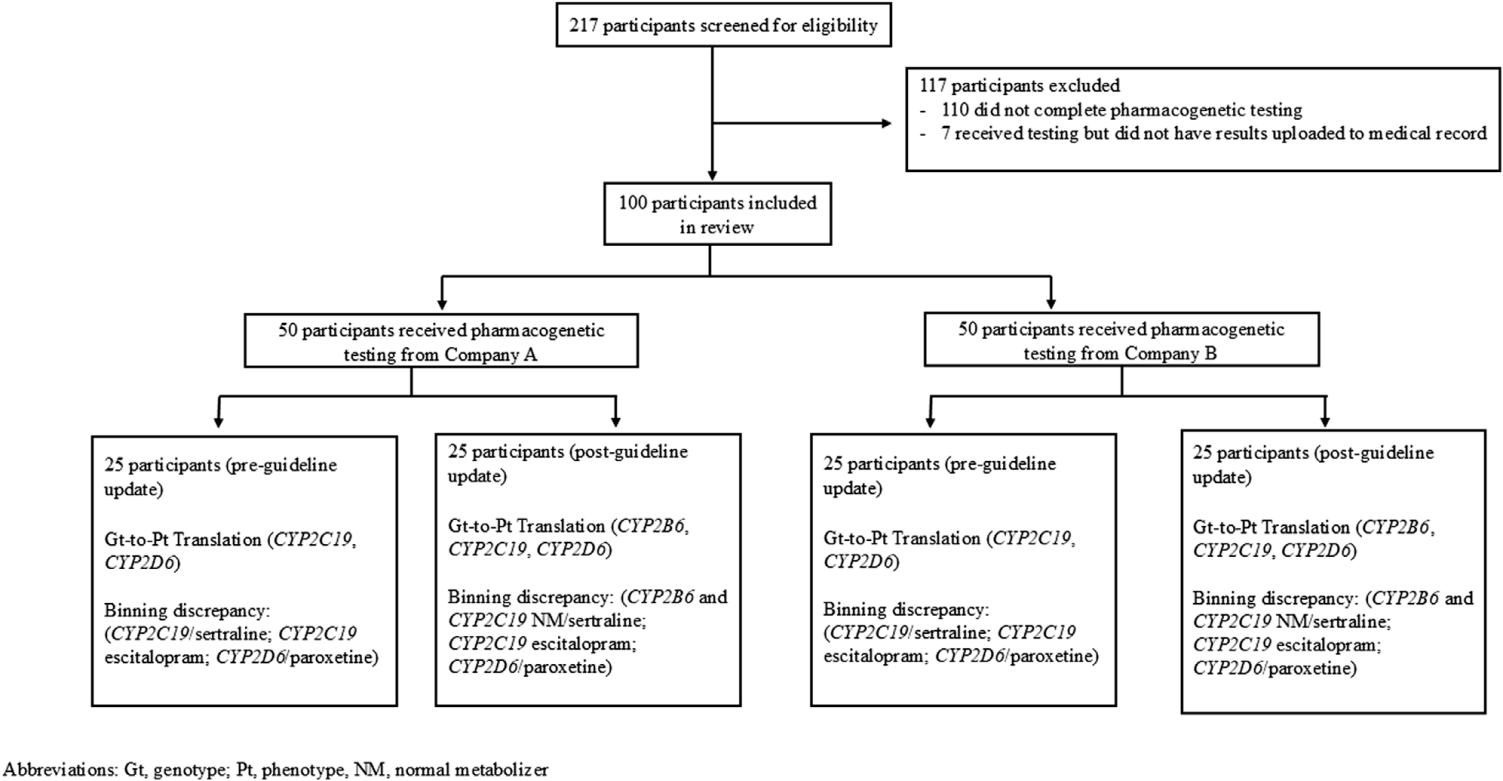
Enrollment.

CPIC published the SSRI guideline in 2015 (Hicks et al., 2015) and it was updated in April 2023 (Bousman et al., 2023). This was taken into consideration when collecting and analyzing data (Figure 1). Due to the updated guideline, patients who had their pharmacogenetic test report date on or prior to 9 April 2023, had to be analyzed separately than those with report dates on or after 10 April 2023. The previous guideline reported only *CYP2C19* impacted sertraline (Hicks et al., 2015), but the updated guideline reported sertraline is impacted by *CYP2C19* and *CYP2B6* (Bousman et al., 2023). Of note, April 10th was chosen as this was the date that PharmGKB implemented the guideline change per their history tab. While the authors recognize that pharmacogenetic testing companies may not be able to immediately update their results to conform with guideline updates, there is no standard timeframe by which companies update their medication recommendation algorithms and thus for simplicity the PharmGKB release date was used.

Genes selected for the evaluation of genotype-to-phenotype translations included *CYP2B6, CYP2C19,* and *CYP2D6,* as both Company A and B report these genes, and tables to support genotype-to-phenotype translations were available per CPIC. For patients who had their pharmacogenetic report date recorded on or before 9 April 2023, the 2015 CPIC guidelines were used and the genotype-to-phenotype translation looked at *CYP2C19* and *CYP2D6*. Medication recommendation evaluations were done with *CYP2D6* and paroxetine as well as both escitalopram and sertraline for *CYP2C19* (Figure 1). For patients reported after the guideline change, the genotype-to-phenotype translation included *CYP2B6, CYP2C19* and *CYP2D6* genes, and drug-gene pairs included *CYP2B6* and sertraline, *CYP2C19* and escitalopram, and *CYP2D6* and paroxetine (Figure 1). While the 2015 CPIC guidelines reported *CYP2D6* affected paroxetine and fluvoxamine, paroxetine was chosen as the medication for *CYP2D6*’s drug-gene pair since it is more commonly prescribed than fluvoxamine at our institution.

### 2.5 Statistical analysis

Data were identified by statistical analysis software (SAS) and evaluated qualitatively. No statistical analysis was done as the objective was to qualitatively assess each company individually and report the findings.

## 3 Results

### 3.1 Evaluated population

While ancestry/ethnic background of the included patients was not collected, the health system at which these patients were tested overwhelmingly serves a population of mostly western European ancestry individuals. Two hundred seventeen patients were screened to obtain a sufficient sampling of patients and 117 of these patients were excluded because they either did not complete pharmacogenetic testing (e.g., an order was placed but the patient did not go through with testing) or did not have their pharmacogenetic results uploaded to their medical record (Figure 1). Of the 100 patients included in the data analysis, 50 patients (25 from each company) received pharmacogenetic testing prior to the updated CPIC guidelines and 50 patients (25 from each company) received pharmacogenetic testing after the updated CPIC guidelines.

### 3.2 Genotype-to-phenotype translation

The 50 patients who received testing prior to the updated CPIC guidelines had two genes reviewed (*CYP2C19* and *CYP2D6*) corresponding to 100 genotype-to-phenotype translations, while the 50 patients who received testing after the updated CPIC guidelines had three genes reviewed (*CYP2B6, CYP2C19*, and *CYP2D6*) corresponding to 150 genotype-to-phenotype translations. In total, 250 genotype-to-phenotype translations were reviewed between both companies and 32 (12.8%) were discordant with CPIC (all from Company A). Of the 32 discrepancies identified, *CYP2C19* had the most discrepancies (n = 17, 53.1%), followed closely by *CYP2D6* (n = 13, 40.6%), and finally *CYP2B6* (n = 2, 6.3%) (Table 3). The most common discrepant genotypes for *CYP2C19* was *1/*17 (seven instances before and seven instances after guideline update), and *2A/*4 for *CYP2D6* (five instances before and three instances after guideline update) for Company A. No discrepancies were found for Company B.

**TABLE 3:** Company genotype-to-phenotype translation comparison discrepancies.

Gene	Genotype	Percentage % (n)	Company phenotype	CPIC phenotype
Company A – 32 discrepancies				
*CYP2B6*	2 discrepancies (6.3%)			
	*1/*4	100 (2)	UM	RM
*CYP2C19*	17 discrepancies (53.1%)			
	*1/*17	82.4 (14)	NM	RM
	*2/*17	17.6 (3)	NM	IM
*CYP2D6*	13 discrepancies (40.6%)			
	*2A/*2A	23.1 (3)	UM	NM
	*2A/*4	61.5 (8)	NM	IM
	*2A/*5	7.7 (1)	NM	IM
	*4/*41	7.7 (1)	PM	IM
Company B – 0 discrepancies				

Abbreviations: UM, ultrarapid metabolizer; RM, rapid metabolizer; NM, normal metabolizer; IM, intermediate metabolizer; PM, poor metabolizer.

### 3.3 Drug-gene pairs evaluation

#### 3.3.1 Overall evaluation

**FIGURE 2:**
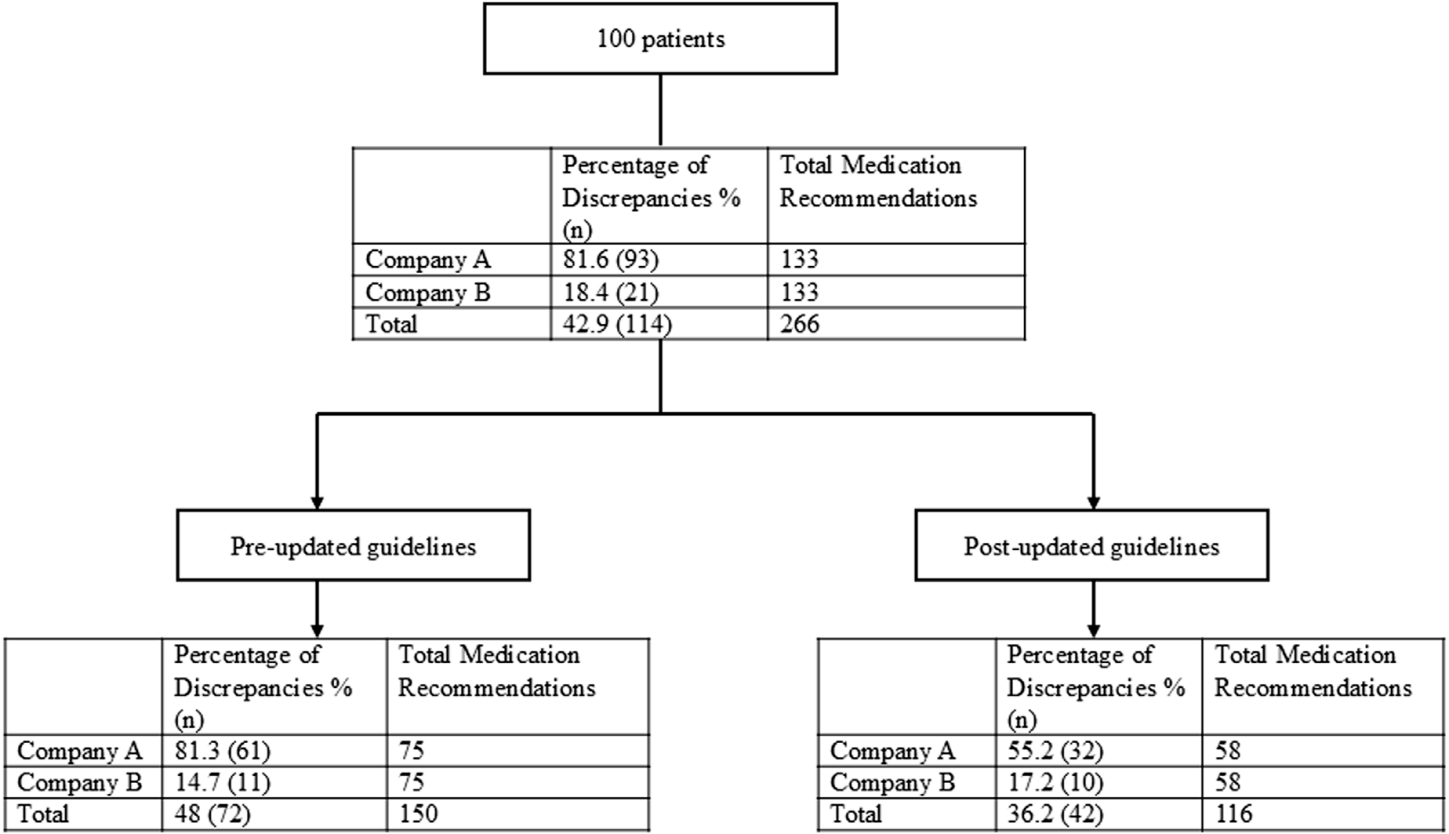
Overall medication recommendation evaluation and results.

In total, within the 100 patients included in the evaluation, 266 drug-gene pairs were reviewed in which 152 (57.1%) were congruent with CPIC’s SSRI guideline and 114 (42.9%) were discrepant (Figure 2). The 25 patients from each company who received testing prior to the guideline update had three drug-gene pairs that were reviewed for a total of 75 drug-gene pairs. For the patients who received testing after the guideline update, to ensure only *CYP2B6* would influence sertraline’s medication recommendations, sertraline recommendations were only considered if the patient was a normal metabolizer for *CYP2C19*. Each company had eight patients who were *CYP2C19* normal metabolizers, and thus had three drug-gene pairs reviewed (*CYP2B6*/sertraline, *CYP2C19*/escitalopram, *CYP2D6*/paroxetine), yielding 24 recommendations. For the 34 patients (17 from each company) that were *CYP2C19* non-normal metabolizers, only *CYP2C19*/escitalopram and *CYP2D6*/paroxetine medication recommendations were considered yielding a total of 34 recommendations. Of these 133 (= 75 + 24 + 34) medication recommendations made by *each* company before and after the guideline update, Company A had 93/133 (70%) that were discrepant with CPIC while Company B had 21/133 (15.8%) (Table 4; Figure 2).

**TABLE 4:** Overall medication recommendation concordance comparison.

	Percentage of congruencies % (n)	Percentage of discrepancies % (n)	Total
Company A	30 (40)	70 (93)	100 (133)
Company B	84.2 (112)	15.8 (21)	100 (133)

#### 3.3.2 Prior to CPIC SSRI guideline update

The 50 patients (25 from Company A, 25 from Company B) who received testing prior to the updated CPIC guidelines had three drug-gene pairs reviewed for medication recommendation comparisons (escitalopram/*CYP2C19*, sertraline*/CYP2C19* and paroxetine*/CYP2D6*), corresponding to 150 drug-gene pairs (75 per company). Out of the 75 medication recommendations made by Company A, 61 (81.3%) were discrepant with the novel binning system (Table 5; Figure 2). Of the discordant medication recommendations from Company A, 22 (36.1%), 22 (36.1%), and 17 (27.8%) were for the drug-gene pairs of escitalopram/*CYP2C19*, paroxetine/*CYP2D6*, and sertraline/*CYP2C19*, respectively (Table 6). The most common genotypes that had medication recommendation discrepancies were CYP2C19*1/*1 and *1/*17 for escitalopram, CYP2D6*1/*4 and *2A/*4 for paroxetine, and CYP2C19*1/*1 for sertraline (Table 6). Company B had 11/75 (14.7%) of their medication recommendations that were discordant which mostly impacted paroxetine and sertraline (Table 5; Figure 2). One escitalopram discrepancy was identified for CYP2C19*1/*17. Paroxetine/*CYP2D6* had 5 discrepancies, of which were two were for *1/*4+*68, two for *2A/*4+*68 and one for *3/*9. Sertraline/*CYP2C19* had 5 discrepancies in total for genotypes *1/*2 and *1/*1 (Table 6).

**TABLE 5:** Medication recommendation comparison for patients receiving testing prior to updated CPIC guideline.

	Percentage of congruencies % (n)	Percentage of discrepancies % (n)	Total
Company A	18.7 (14)	81.3 (61)	100 (75)
Company B	85.3 (64)	14.7 (11)	100 (75)

**TABLE 6:** Frequency of genotypes with discrepant medication recommendations for patients receiving testing prior to updated CPIC guidelines.

Drug-gene pair	Genotype	Percentage % (n)
Company A – 61 discrepancies		
Escitalopram and *CYP2C19*	22 discrepancies	
	*1/*1	45.5 (10)
	*1/*17	31.8 (7)
	*1/*2	13.6 (3)
	*2/*17	9.1 (2)
Paroxetine and *CYP2D6*	22 discrepancies	
	*1/*1	9.1 (2)
	*1/*2A	13.7 (3)
	*1/*3	9.1 (2)
	*1/*4	18.2 (4)
	*1/*41	4.5 (1)
	*1/*5	4.5 (1)
	*2A/*2A	9.1 (2)
	*2A/*4	18.2 (4)
	*2A/*41	4.5 (1)
	*2A/*9	9.1 (2)
Sertraline and *CYP2C19*	17 discrepancies	
	*1/*1	58.9 (10)
	*1/*17	5.9 (1)
	*1/*2	17.6 (3)
	*17/*17	5.9 (1)
	*2/*17	11.7 (2)
Company B – 11 discrepancies		
Escitalopram and *CYP2C19*	1 discrepancy	
	*1/*17	100 (1)
Paroxetine and *CYP2D6*	5 discrepancies	
	*1/*4+*68	40 (2)
	*2A/*4+*68	40 (2)
	*3/*9	20 (1)
Sertraline and *CYP2C19*	5 discrepancies	
	*1/*1	40 (2)
	*1/*2	60 (3)

#### 3.3.3 Following the CPIC SSRI guideline update

Of the 50 patients who were tested following the guideline update posted on the PharmGKB website, 16 patients (8 from Company A and 8 from Company B) were normal metabolizers for *CYP2C19* and had 3 drug-gene pairs (sertraline/*CYP2B6*, escitalopram/*CYP2C19*, and paroxetine/*CYP2D6*) reviewed, corresponding to 48 drug-gene pairs. The other 34 patients (17 from Company A and 17 from Company B) who were non-normal metabolizers for *CYP2C19* had 2 drug-gene pairs (escitalopram/*CYP2C19* and paroxetine/*CYP2D6*) reviewed, corresponding to 68 drug-gene pairs. There were 116 (58 from both companies) total medication recommendations reviewed for patients who had their pharmacogenetic test report date after the updated guidelines. Company A had 32/58 (55.2%) of their recommendations that were discrepant, while Company B had 10/58 (17.2%) that were discordant (Table 7; Figure 2). Company A had a majority of the discrepant recommendations for *CYP2C19*/escitalopram and *CYP2D6*/paroxetine, 15/32 (46.9%) and 14/32 (43.8%) respectively (Table 8). Many medication discrepancies for escitalopram and *CYP2C19* were for the genotypes of *1/*1 and *1/*2, while paroxetine and *CYP2D6* had more had discordant medication recommendations for *1/*2A and *2A/*4 (Table 8). Company B had 8/10 (80%) discrepancies related to escitalopram and *CYP2C19* and 2/10 (20%) were related to sertraline and *CYP2B6* (Table 8). The genotypes that were discordant for escitalopram and *CYP2C19* were *1/*17 and *1/*2, and CYP2B6*1/*6 for sertraline.

**TABLE 7:** Medication recommendation comparison for patients receiving testing after updated CPIC guidelines.

	Percentage of congruencies % (n)	Percentage of discrepancies % (n)	Total
Company A	44.8 (26)	55.2 (32)	100 (58)
Company B	82.8 (48)	17.2 (10)	100 (58)

**TABLE 8:** Frequency of genotypes with discrepant medication recommendations for patients receiving testing after updated CPIC guidelines.

Drug-gene pair	Genotype	Percentage % (n)
Company A – 32 discrepancies		
Escitalopram and *CYP2C19*	15 discrepancies	
	*1/*1	53.3 (8)
	*1/*17	13.3 (2)
	*1/*2	26.7 (4)
	*17/*17	6.7 (1)
Paroxetine and *CYP2D6*	14 discrepancies	
	*1/*2A	21.5 (3)
	*1/*4	7.1 (1)
	*1/*41	14.4 (2)
	*2/*2A	7.1 (1)
	*2A/*10	7.1 (1)
	*2A/*2A	7.1 (1)
	*2A/*4	21.5 (3)
	*2A/*5	7.1 (1)
	*4/*41	7.1 (1)
Sertraline and *CYP2B6*	3 discrepancies	
	*1/*1	66.7 (2)
	*1/*4	33.3 (1)
Company B – 10 discrepancies		
Escitalopram and *CYP2C19*	8 discrepancies	
	*1/*17	50 (4)
	*1/*2	50 (4)
Paroxetine and *CYP2D6*	0 discrepancies	
Sertraline and *CYP2B6*	2 discrepancies	
	*1/*6	100 (2)

#### 3.3.4 Variable binning for the same genotype and drug-gene pair

Interestingly, there were 18 instances where companies had different medication recommendations for the same genotype for the same drug-gene pair among different patients (Supplementary Table A). This occurred 17 times for Company A and once for Company B. For Company A, *CYP2C19* and *CYP2D6* were impacted: there were three instances for CYP2C19*1/*17 that had binned escitalopram as “minimal/limited” DGI, but for the other instances the genotype/drug-gene pair was binned as “moderate”. Additionally, escitalopram/*CYP2C19* *1/*2 was binned as “moderate” three times but were categorized as “major” in the remainder of cases. Paroxetine/*CYP2D6* had considerable variability for genotypes *1/*2A, *1/*3, *1/*4, and *1/*41 in which about half of the cases for each genotype were binned ‘moderate’, and the other half were binned “major”. Remarkably, CYP2D6 *2A/*4 had two instances when it was binned as “major”, one instance when it was binned as “minimal/limited” DGI, and the remaining instances when it was binned as “moderate”. Company B only had one occurrence for *CYP2C19* *1/*17 and escitalopram where they placed the DGI under “moderate” interaction, but in all other identified cases it was in the “major” interaction category.

#### 3.3.5 Trends in “level of impact” of binning

Of the 114 medication recommendation discrepancies, 95 (83.3%; Company A = 81, Company B = 14) of these were binned with a higher level of impact compared to the novel binning system based upon CPIC recommendations, while 19 (16.7%; Company A = 12, Company B = 7) were binned with a lower level of impact (Supplemental Table B). Stated differently, in 95 cases the company-reported bin indicated a DGI with a higher level of impact compared to the novel bin designation. In 19 cases, the opposite was true, whereas the CPIC-based bin indicated a higher level of impact compared to the company-reported bin.

## 4 Discussion

Our data confirms previously reported discrepancies between pharmacogenetic testing companies’ translation of genotype-to-phenotype with significant impact on *CYP2C19* and *CYP2D6* (Blazy et al., 2022). Furthermore, our findings support the logical outcome of that discrepancy: divergence in the genotype-to-phenotype translation will yield divergence in medication recommendations between the testing company and CPIC recommendations. These discrepancies are interpreted through the lens of a novel binning process; however, the binning process was designed to align with CPIC’s recommendations based upon consistent language and trends in recommended action(s) used by CPIC in regard to which bin the recommendation would fall. With that acknowledgement, the results from our study showed that there were substantial discrepancies between testing companies’ interpretations and medication recommendations compared to CPIC.

The use of proprietary algorithms not only yields discrepancies between testing companies and professional resources such as CPIC, but also discrepancies from company to company. In a study that compared four different commercial pharmacogenetic testing companies amongst each other, only *CYP2C9* out of seven genes had 100% congruency in the genotype-to-phenotype translation (Bousman and Dunlop, 2018). Three genes saw perfect genotype congruency (*CYP2C19*, *CYP3A4*, and *UGT2B15*), but reported different translated phenotypes ranging from 33% to 89% congruency (Bousman and Dunlop, 2018). Only 58% of the medication recommendations were in agreement among the testing companies while 71% of all genotypes were in agreement.

Further, a study by Blazy et al. evaluating one testing company found that *CYP2D6* and *CYP2C19*’s genotype-to-phenotype translation was discrepant 28.8% and 32.2% respectively when compared to CPIC’s standards (Blazy et al., 2022). The article found that common differences for *CYP2D6* were *2A/*2A, *2A/4, and *4/*41, and for *CYP2C19* were *1/*17 (Blazy et al., 2022). All of these were consistent with the results found in this study. With the aforementioned incongruencies of pharmacogenetic testing companies’ interpretations, transparency in how the laboratories translate genotypes to phenotypes, and phenotypes to medication recommendations is crucial for implementers and clinical users of pharmacogenetics to know what these recommendations are based upon (Vo et al., 2017).

To the best of our knowledge, this is the first time that a study has reported companies making different medication recommendations for the same genotype and drug-gene pair (Supplemental Table A). This occurred multiple times for escitalopram and paroxetine. For the majority of these instances the Companies binned these medications in the moderate (yellow) or major (red) category. The use of proprietary algorithms may explain the different categorization by including additional genes to advise on medication response, though notably these algorithms are not in-line with CPIC recommendations, and may lead providers to exclude a medication that may otherwise benefit a patient. In the mental health realm, inappropriately excluding a therapeutic alternative could cause unwarranted escalation in therapy which may result in additional costs, side effects, and monitoring for the patient. For many providers, paroxetine and escitalopram are familiar medications with generally favorable tolerance and side effect profiles, thus standardization of pharmacogenetic recommendation practices is vital to ensure proper use of test results. Ultimately, the identified discrepancies in medication recommendations could lead to provider confusion and distrust with pharmacogenetics, and further demonstrates the potential harm of proprietary algorithms used by commercial vendors and the importance of standardization in the pharmacogenetics field.

Medication recommendation discrepancies are not unique to comparisons between testing companies and professional guidelines as differences in medication recommendations have also been described between multiple government (e.g., FDA) and/or professional guidelines (Bank et al., 2018; Pritchard et al., 2022; Shugg et al., 2020). However, professional guidelines generally have transparency in their guideline development process (Caudle et al., 2014), and it is understood that the FDA has access to data provided by drug manufacturers that may not be available to the general public. Further, guideline development processes generally have policies and safeguards against potential or actual conflicts of interest, and medication recommendation discrepancies between organizations/guidelines are often explained by differences in the scope/perspectives, resources, and time-effect of the recommendations (Pritchard et al., 2022).

Binning of drug interactions is not a novel concept as it is used widely by a variety of professional pharmacotherapy drug interaction checking resources. As this work aimed to build upon previous studies in terms of comparison of testing company recommendations to CPIC’s guidelines, a means of categorization for comparison with the testing companies was necessary. Users may find the simple color-coded binned recommendations easy to use and appealing for making a prompt determination in medication selection. Thus, to resolve the dilemma of medication recommendation comparison, a binning system was created to adhere as faithfully as possible to CPIC recommendations. It is prudent to acknowledge that a variety of opinions and relevant cautions exist regarding the binning systems (Dunlop, 2020) as the proprietary algorithms used to support these binning systems may leave room for conflicts of interest in the science. It is not uncommon to see genes on panels that do not currently have CPIC guidelines as “more genes and/or drugs” may be perceived by some as constituting a ‘better’ pharmacogenetic report. The authors also acknowledge limitations of binning systems including color-schemes that are not friendly to colorblind users as well as the oversimplification of the pharmacogenetic recommendations implied with binning, which could lead clinicians to avoid an appropriate medication that would simply require dose modification or monitoring based upon pharmacogenetics. Should binning of medication recommendations become an accepted practice among the pharmacogenetic community, standardization of binning practices is one area that may improve pharmacogenetic awareness, uptake, and utilization.

There were some limitations to this real-world evaluation. The sample size was relatively small, and from a single rural health system with unique features of pharmacogenetic integration into the medical record (including clinical decision support integration of Company B into the medical record). This work covered a 5-year period during which significant growth in technology and testing practice changes occurred. As such, the testing platforms used by the companies may have had changes (e.g., changes in methodology, variant coverage, etc.) that were not accounted for in the analysis. Further, during the queried timeframe the health system changed electronic medical record vendors and ordering practices which may have impacted identification of certain patients during the timeframe of 2021–2023. As which specific resource(s) Company A and Company B rely heavily upon to make their medication recommendations are not publicly available, the assumption of the relevance in timing of the CPIC SSRI guideline update may or may not actually have been impactful for analysis for these companies. If it was impactful, it is important to note it may take time for testing companies to respond to CPIC guideline updates (assuming they update their algorithm based upon this data), as such records that were collected in and around April 2023 may not be truly representative of the company’s practices at that time. Of note, CPIC members are often notified of planned guideline updates prior to the publication of the guideline; thus, if the pharmacogenetic testing company had data curators who were also CPIC members, they may have been able to anticipate incorporation of the updated guidelines to their interpretations and medication recommendations. The authors also recognize several reputable resources for pharmacogenetic interpretation exist besides CPIC, however to simplify the comparison for this project, only CPIC was utilized for the novel binning system.

In conclusion, discrepancies from CPIC guidelines were identified for genotype-to-phenotype translations and medication recommendations, including variable medication recommendations for the same genotype and drug-gene pairs within the same company. These findings are concerning as proprietary practices by the testing companies may lead to providers making medication recommendations that are not supported by clinical practice guidelines, which in turn could lead to suboptimal outcomes for patients and result in both patient and provider dissatisfaction with pharmacogenetic testing. A proposed resolution for the discrepancies in company-to-company interpretation is adherence to the CPIC guidelines. Further work in this area may benefit from similar comparison between testing companies and the FDA resources such as the Table of Pharmacogenetic Associations.

## Data Availability

The datasets presented in this article are not readily available because the data contains patient specific information, specifically genotype, phenotype and medication recommendation results. Requests to access the datasets should be directed to the corresponding author.
