# Supplementary material for: Comparing commercial pharmacogenetic testing results and recommendations for antidepressants with established CPIC guidelines
Source: Front Pharmacol. 2024 Nov 25;15:1500235. doi: 10.3389/fphar.2024.1500235 (PMC11626124; doi:10.3389/fphar.2024.1500235)
Supplement: Supplementary file 1 [file Table1.docx]

Supplementary Material

**Table A. Occurrences of different medication recommendations for the same genotype of the same drug gene pair**

| **Drug-gene pair** | **Genotype** | **Company phenotype** | **Most common bin used** | **Other bin identified for the same pair** | **Frequency of discrepant bins for same pair** |
| --- | --- | --- | --- | --- | --- |
| **Company A** | | | | | |
| Escitalopram and *CYP2C19* | *1/*17 | NM | Moderate | Minimal/Limited | 3 |
|  | *1/*2 | IM | Major | Moderate | 3 |
| Paroxetine and *CYP2D6* | *1/*2A | NM | Moderate | Major | 3 |
|  | *1/*3 | IM | Moderate | Major | 1 |
|  | *1/*4 | IM | Moderate | Major | 2 |
|  | *1/*41 | IM | Moderate | Major | 1 |
|  | *2A/*4 | NM | Moderate | Major | 2 |
|  | *2A/*4 | NM | Moderate | Minimal/limited | 1 |
|  | *2A/*9 | NM | Moderate | Major | 1 |
| **Company B** | | | | | |
| Escitalopram and *CYP2C19* | *1/*17 | RM | Major | Moderate | 1 |

Abbreviations: RM, rapid metabolizer; NM, normal metabolizer; IM, intermediate metabolizer

**Table B. Comparison of company and CPIC recommendation level of impact**

|  | **% Company more severe (n)** | **% Company less severe (n)** |
| --- | --- | --- |
| Company A | 85.3 (81) | 63.2 (12) |
| Company B | 14.7 (14) | 36.8 (7) |
| Total | 100 (95) | 100 (19) |
